# Supplementary material for: Causal Relationship between Aging and Anorexia Nervosa: A White-Matter-Microstructure-Mediated Mendelian Randomization Analysis
Source: Biomedicines. 2024 Aug 16;12(8):1874. doi: 10.3390/biomedicines12081874 (PMC11351342; doi:10.3390/biomedicines12081874)
Supplement: Supplementary file 1 [file biomedicines-12-01874-s001.zip › Supplementary Figure/Figure S4/Singe SNP plot of MR analysis for TL to ubm-b-2016.pdf]

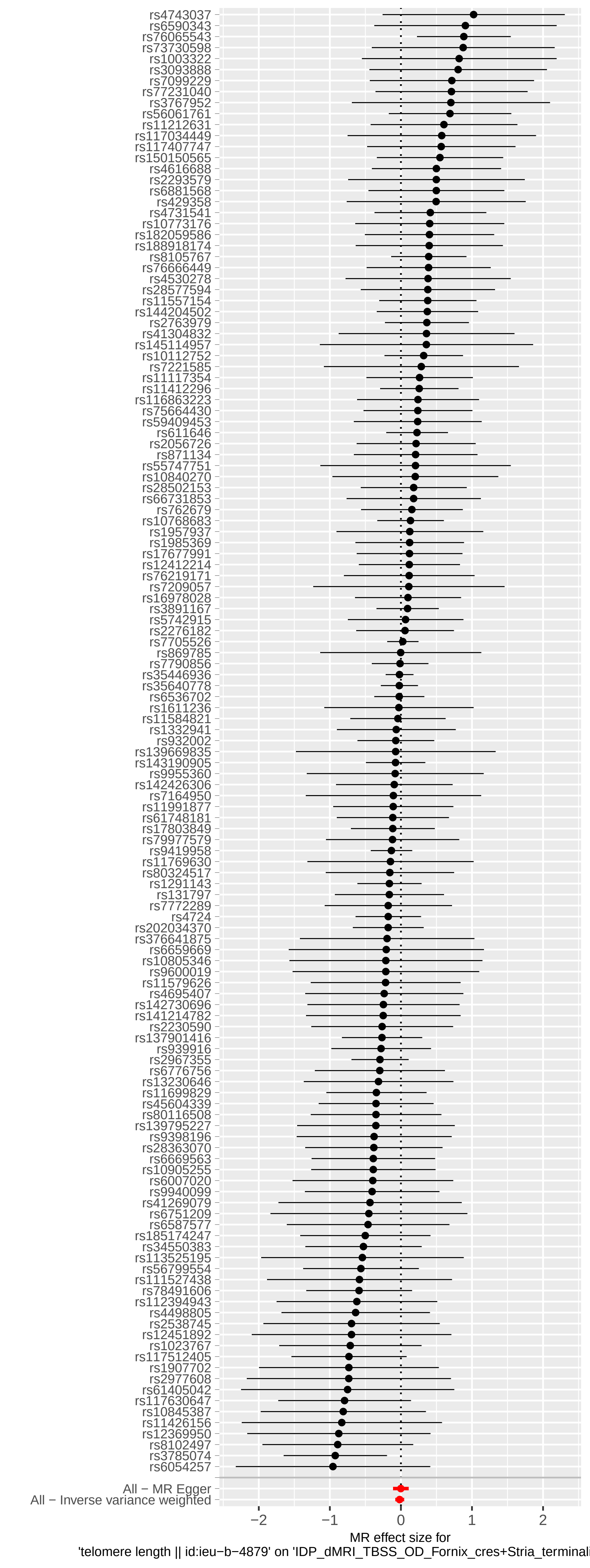
rs4743037
rs6590343
rs76065543
rs73730598
rs1003322
rs3093888
rs7099229
rs77231040
rs3767952
rs56061761
rs11212631
rs117034449
rs117407747
rs150150565
rs4616688
rs2293579
rs6881568
rs429358
rs4731541
rs10773176
rs182059586
rs188918174
rs8105767
rs76666449
rs4530278
rs28577594
rs11557154
rs144204502
rs2763979
rs41304832
rs145114957
rs10112752
rs7221585
rs11117354
rs11412296
rs116863223
rs75664430
rs59409453
rs611646
rs2056726
rs871134
rs55747751
rs10840270
rs28502153
rs66731853
rs762679
rs10768683
rs1957937
rs1985369
rs17677991
rs12412214
rs76219171
rs7209057
rs16978028
rs3891167
rs5742915
rs2276182
rs7705526
rs869785
rs7790856
rs35446936
rs35640778
rs6536702
rs1611236
rs11584821
rs1332941
rs932002
rs139669835
rs143190905
rs9955360
rs142426306
rs7164950
rs11991877
rs61748181
rs17803849
rs79977579
rs9419958
rs11769630
rs80324517
rs1291143
rs131797
rs7772289
rs4724
rs202034370
rs376641875
rs6659669
rs10805346
rs9600019
rs11579626
rs4695407
rs142730696
rs141214782
rs2230590
rs137901416
rs939916
rs2967355
rs6776756
rs13230646
rs11699829
rs45604339
rs80116508
rs139795227
rs9398196
rs28363070
rs6669563
rs10905255
rs6007020
rs9940099
rs41269079
rs6751209
rs6587577
rs185174247
rs34550383
rs113525195
rs56799554
rs111527438
rs78491606
rs112394943
rs4498805
rs2538745
rs12451892
rs1023767
rs117512405
rs1907702
rs2977608
rs61405042
rs117630647
rs10845387
rs11426156
rs12369950
rs8102497
rs3785074
rs6054257
All − MR Egger
All − Inverse variance weighted
−2
−1
0
1
2
MR effect size for
'telomere length || id:ieu−b−4879' on 'IDP_dMRI_TBSS_OD_Fornix_cres+Stria_terminali
